# BlsA Is a Low to Moderate Temperature Blue Light Photoreceptor in the Human Pathogen *Acinetobacter baumannii*

**DOI:** 10.3389/fmicb.2019.01925

**Published:** 2019-08-21

**Authors:** Adrián E. Golic, Lorena Valle, Paula C. Jaime, Clarisa E. Álvarez, Clarisa Parodi, Claudio D. Borsarelli, Inés Abatedaga, María Alejandra Mussi

**Affiliations:** ^1^Centro de Estudios Fotosintéticos y Bioquímicos (CEFOBI-CONICET), Universidad Nacional de Rosario (UNR), Rosario, Argentina; ^2^Instituto de Bionanotecnología del NOA (INBIONATEC) CONICET-Universidad Nacional de Santiago del Estero (UNSE), Santiago del Estero, Argentina

**Keywords:** *Acinetobacter baumannii*, BLUF, motility, photoactivity, temperature modulation

## Abstract

Light is an environmental signal that produces extensive effects on the physiology of the human pathogen *Acinetobacter baumannii*. Many of the bacterial responses to light depend on BlsA, a bluelight using FAD (BLUF)-type photoreceptor, which also integrates temperature signals. In this work, we disclose novel mechanistic aspects of the function of BlsA. First, we show that light modulation of motility occurs only at temperatures lower than 24°C, a phenotype depending on BlsA. Second, *blsA* transcript levels were significantly reduced at temperatures higher than 25°C, in agreement with BlsA protein levels in the cell which were undetectable at 26°C and higher temperatures. Also, quantum yield of photo-activation of BlsA (lBlsA) between 14 and 37°C, showed that BlsA photoactivity is greatly compromised at 25°C and absent above 28°C. Fluorescence emission and anisotropy of the cofactor together with the intrinsic protein fluorescence studies suggest that the FAD binding site is more susceptible to structural changes caused by increments in temperature than other regions of the protein. Moreover, BlsA itself gains structural instability and strongly aggregates at temperatures above 30°C. Overall, BlsA is a low to moderate temperature photoreceptor, whose functioning is highly regulated in the cell, with control points at expression of the cognate gene as well as photoactivity.

## Introduction

Blue light modulates multiple cellular processes in the important human pathogen *Acinetobacter baumannii* including metabolic pathways such as the phenylacetic acid (PAA) degradation pathway and trehalose biosynthesis, tolerance to some antibiotics as well as antioxidant enzyme levels such as catalase. These traits likely contribute to the bacterial persistence in adverse environments (Müller et al., 2017). Moreover, the expression of whole pathways and gene clusters, such as genes involved in lipid metabolism, the complete type VI secretion system, as well as efflux pumps related to antibiotic resistance, are differentially induced by blue light (Müller et al., 2017). In previous work, we further showed that in *A. baumannii*, blue light also modulates motility, biofilm formation, and virulence against *Candida albicans* (Mussi et al., 2010).

Most of these blue light-depending processes are governed by BlsA, a Blue Light Using FAD (BLUF) photoreceptor. BlsA is therefore a global regulator, capable of modulating different cellular processes simultaneously in a light-dependent way (Mussi et al., 2010; Müller et al., 2017; Tuttobene et al., 2018). Recently, we have demonstrated that BlsA directly interacts and antagonizes the functioning of diverse transcriptional regulators as the ferric uptake regulator (Fur) in the dark, enabling expression of the acinetobactin siderophore gene cluster, as well as growth under iron deprived conditions (Tuttobene et al., 2018). Also, BlsA interacts with and antagonizes the functioning of the acetoin catabolism repressor, AcoN, but only under blue light (Tuttobene et al., 2019b). In the last years only a few combinations of BLUF domains and protein partners have been characterized; e.g., photosynthesis-related gene expression in the purple bacterium *Rhodobacter sphaeroides* is controlled by AppA-PpsR (Pandey et al., 2011), biofilm formation in *Escherichia coli* by YcgF-YcgE (Tschowri et al., 2009) and the phototaxis response in the cyanobacterium *Synechocystis* sp. PCC6803 by PixD–PixE (Fujisawa and Masuda, 2018). However, BlsA is the only so far shown to function as a global regulator.

Therefore, getting insight into the mechanism of BlsA function would provide clues regarding photoregulation in *A. baumannii*, which governs important traits related to bacterial persistence in the environment and virulence. In addition, this knowledge would enlarge our understanding of short BLUFs, which represent most of the BLUF-domain containing proteins present in bacterial genomes. Short BLUFs harbor only a short amino acidic extension of approximately 50 amino acids after the BLUF domain with no significant homology (van der Horst and Hellingwerf, 2007). Indeed, the absence of an output domain within the same molecule is overcome by interacting with other protein partner/s leading to signal transduction to multiple effectors in the case of BlsA, thus explaining its nature as a global regulator (Gomelsky and Hoff, 2011). We have previously demonstrated that BlsA also integrates temperature signals in addition to the blue-light input (Mussi et al., 2010; Abatedaga et al., 2017). Indeed, photoregulation of motility occurs at 24°C but not at 37°C in wild type cells (Mussi et al., 2010; Wood et al., 2018). Concomitantly, *blsA* expression and photoreceptor levels were significantly reduced at 37°C when compared to 24°C, likely accounting for the absence of photoregulation at this temperature (Abatedaga et al., 2017). In this sense, the *E. coli* photoreceptor YcgF, a fosfodiesterase which harbors a BLUF domain as a sensor in its architecture (Hasegawa et al., 2006; Nakasone et al., 2007; Tschowri et al., 2009), has also been proposed to be a blue light photoreceptor temperature sensitive (Nakasone et al., 2010). Another point of control that governs the functionality of BlsA is at the photocycle level, since spectroscopic analysis indicated that the protein looses all functionality above 30°C (Abatedaga et al., 2017). Most likely the displacement of the FAD cofactor from the adequate orientation within its pocket alters the prompt photoinduced electron-transfer (ET) coupled with a fast proton transfer with the conserved Tyr7 (Dragnea et al., 2005; Gauden et al., 2006; Mathes et al., 2012). Moreover, even under dark conditions, a fraction of the protein precipitated above 30°C, probably by massive BlsA aggregation due to drastic conformational modification. We hypothesized that external and internal conformational changes driven by temperature must occur in BlsA, since the activation energies for the formation of the light-activated state and its relaxation back to the dark form (lBlsA and dBlsA, respectively) follows an enthalpy-entropy compensation effect (Abatedaga et al., 2017), probably due to the breakdown of hydrogen-bonding interactions between the cofactor and surrounding residues in the FAD pocket. In fact, structural modifications in BlsA were also demonstrated by vibrational studies proving that the C2=O of FAD loses hydrogen-bonding interactions with the concomitant weakening of BlsA β-sheet upon illumination, with significant differences in the β5 strand (Brust et al., 2014).

In this work, we have investigated the influence of temperature on photoregulation mediated by BlsA in *A. baumannii*, by determining the critical temperature of BlsA functioning through monitoring a phenotype that depends on BlsA such as motility (Mussi et al., 2010; Abatedaga et al., 2017; Wood et al., 2018). A critical temperature for photoregulation of motility of 25 ± 1°C was determined, consistent with a significantly reduced *blsA* expression as well as photoreceptor levels in the cells at higher temperatures. In particular, we were able to detect photoregulation of motility at 18, 21, 23, and 24°C while at 25, 26, 27, 28, 30, and 35°C the photoregulation was lost, indicating 24°C being a critical temperature. Second, *blsA* expression levels were significantly reduced at temperatures higher than 25°C such as 26 or 30°C, while at 21 and 23°C expression of *blsA* was significant, with higher levels observed in the dark rather than under blue light. These results are in agreement with BlsA levels in the cell, which were undetectable from 26°C on. Furthermore, the critical temperature in which BlsA has the ability to form the light-activated state was determined to be 25°C, confirming that this photoreceptor experiences sufficient structural compromises in the FAD nanocavity so that its ability to respond to blue light is affected. Finally, the quaternary structure of BlsA and the dynamic changes of its aggregation with temperature confirmed that a small fraction of soluble protein is still present in solution, despite precipitation and loss of activity observed for the rest. The overall data indicate that BlsA is a low to moderate temperature light-sensor, which governs the response to light at low to moderate temperatures in the human pathogen *A. baumannii*.

## Materials and Methods

### Blue Light Treatments

Blue light treatments were performed as described previously (Mussi et al., 2010; Golic et al., 2013; Müller et al., 2017). Briefly, cells were incubated as specified in the dark or under blue light emitted by 9-light-emitting diode (LED) arrays, with an intensity of 6 to 10 μEinsteins m^–2^ s^–1^. Each array was built using 3-LED module strips emitting blue light.

### Motility Assays

These experiments were performed as described previously (Mussi et al., 2010). Briefly, the plates were inoculated on the surface with bacteria lifted from overnight LB (Sambrook and Russell, 2001) agar cultures using flat-ended sterile wooden sticks. In these assays, pairs of plates (one in the dark and the other under blue light) were analyzed, at any given temperature. Plates were incubated until the cells of at least one of the plates of the analyzed pair just reached the edge of the dish at each given temperature. The area of plates covered with bacteria was measured with ImageJ (NIH), and then the percentage of plate coverage was calculated. Three independent experiments were performed for each condition.

### Analyses of Gene Expression by qRT-PCR

Reverse transcription and qRT-PCR analysis were performed as described in references (Müller et al., 2017; Tuttobene et al., 2018), using *blsA* primers described in Müller et al. (2017). Data are presented as NRQ (Normalized relative quantities) calculated by the qBASE method (Hellemans et al., 2007), using *recA* and *rpoB* genes as normalizers (Müller et al., 2017). For motility assays, pairs of plates (one in the dark and the other under blue light) were analyzed, at any given temperature. Cells were harvested from complete plates, at time points in which the cells of at least one of the plates of the analyzed pair just reached the edge of the dish at each given temperature. Three biological replicates were used for each condition.

To analyze the variation of *blsA* transcripts in the short term as a result of light or temperature changes, ATCC^®^ 17978 cells were grown until OD = 0.5 in LB at 24°C in the dark (D) (*t* = 0), and then switched to light conditions, or rather, the cells were grown until OD = 0.5 in LB in the dark (D) at 24°C (*t* = 0), and then switched to 37°C (Mussi et al., 2010). Three biological replicates were used for each condition.

### Protein Analyses

Protein extraction, quantification, SDS-PAGE separation and western blots were performed as indicated in Abatedaga et al. (2017). Recombinant BlsA was produced and purified also as performed in Abatedaga et al. (2017). Western blots were repeated at least twice using biological replicates.

### Quantum Yield of Photoactivation (dBlsA + *h*ν → lBlsA)

Light adapted state for BlsA (lBlsA) was obtained by blue light irradiation of the dark-adapted form (dBlsA) using a 1 W Royal Blue LED (Luxeon Star Leds) at 443 ± 20 nm. The apparent quantum yield of formation of lBlsA state (Φ_lBlsA_) was estimated following a methodology previously described (Abatedaga et al., 2017). Briefly, for each analyzed temperature, Φ_lBlsA_ was calculated using Eq. (1), where *C*_*lBlsA*_ the concentration of lBlsA determinated from the absorbance plateau at 510 nm of the growth kinetic profiles, using Δε_510_ = 3800 M^–1^ cm^–1^ profiles, and *q*_*n,p*_ is the photon flux, determined by chemical actinometry using potassium ferrioxalate (Fukushima et al., 2005; Abatedaga et al., 2017).

(1) $$\Phi_{\text{lBlsA}}=C_{l\,B\,l\,s\,A}\,/\,q_{n,p}$$

### Spectroscopic Measurements

UV-vis absorption spectra were registered using a modular miniature spectrophotometer USB2000+ (Ocean Optics, United States) connected via optic fiber to UV-vis light source (Analytical Instruments System, United States), by placing 300 μL of BlsA in buffer *TRIS* (2-Amino-2-(hydroxymethyl)propane-1,3-diol) 20 mM, NaCl 500 mM, pH 8 in a 5 × 5 mm quartz cuvettes, mounted on a Peltier-driven temperature-controlled cuvette holder (Flash 300 of Quantum Northwest) (Abatedaga et al., 2017).

Emission spectra of the cofactor or the protein were obtained with Hitachi F-2500 spectrofluorometer by selective excitation at 460 nm or 295 nm, respectively. Steady state fluorescence anisotropy *r* was determined using the classical L-format and calculated as previously described (Villegas et al., 2014).

Time-resolved fluorescence anisotropy *r*(*t*) of the cofactor emission of BlsA as a function of the temperature was calculated by measuring the fluorescence emission decays using a Tempro-01 time-correlated single photon counting (TCSPC) system (Horiba, Glasgow), by ultrafast excitation with a 1 MHz Nanoled^®^ emitting at 460 (±20) nm. Fluorescence decays were registered at 510 and 540 nm with an emission bandwidth of ±12 nm (Valle et al., 2015). The fluorescence anisotropy decays were analyzed with the classical exponential model function for a spherical emitter, Eq. (2), where *r*_0_ is the maximum anisotropy at *t* = 0, and θ is the rotational correlation time of the sphere.

(2) $$r\,\left(t\right)=r_{0}\,\exp\left(-t/\theta \right)$$

All fluorescence measurements were performed under air-saturated conditions and controlled temperature at (*T* ± 0.1°C) using an external thermostat (Haake F3, Germany).

### Dynamic Light Scattering Measurements

Dynamic light scattering (DLS) experiments were carried out on a Particle Analyzer SZ100 (Horiba) with a backscattering detection at 90°, using 5 × 5 mm quartz cuvettes. BlsA solutions were left equilibrating for 10 min at each temperature before measurements were performed. The hydrodynamic diameter value (HD) was calculated from a set of 3 measurements (∼40 runs each). The HD of the sample was obtained from the peak with the highest scattered light intensity (i.e., the mode) in number intensity distributions after averaging all runs using the instruments software (SZ-100, Horiba) (Su et al., 2008).

Working buffer solution was filtered with HV (Durapore) PVDF (polyvinylidene difluoride) membranes, 0.45 μm pore size. Purified BlsA was desalted against filtered buffer using a PD-minitrap column pre-equilibrated with 20 volumes of filtered buffer. Last elution of equilibration was used as blank to determine artifacts present in the solution with DLS. Controls with air and buffer-only were performed to detect any possible artifacts. BlsA in working buffer (10 mM phosphate, 10 mM NaCl, pH 7) was used for all the determinations at a protein concentration of 10 μM as calculated using the absorbance at 460 with an ε = 11300 M^–1^ cm^–1^.

## Results

### *Acinetobacter baumannii* Motility Is Regulated by Light Within a Low to Moderate Temperature Range

**FIGURE 1:**
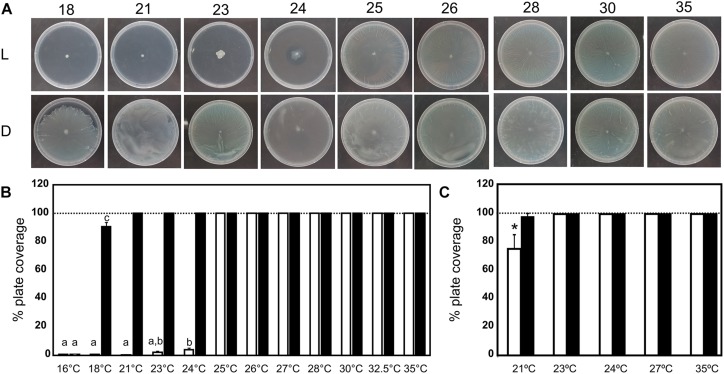
Effects of blue light and temperature on *A. baumannii* motility. **(A)** Cells of the parental strain ATCC 17978 were inoculated on the surface of motility plates and incubated at the indicated temperatures. Plates were inspected and photographed after incubation in darkness (D) or in the presence of blue light (L) at the indicated temperatures. **(B)** Quantification of cell motility estimated as the percentage of plate coverage, i.e., the percentage of the Petri plate area covered with bacteria, in motility plates inoculated with ATCC 17978 wild type and incubated at the indicated temperatures. Three independent experiments were performed in each case. The area of plates covered with bacteria was measured with ImageJ (NIH), and then the percentage of plate coverage was calculated. The mean ± SEM is informed. Different letters indicate significant differences as determined by ANOVA followed by Tukey’s multiple comparison test (*p* < 0.05). For those conditions at which the bacteria just reached the edge of the plate a value of 100% is informed. **(C)** Quantification of cell motility estimated as the percentage of plate coverage, i.e., the percentage of the Petri plate area covered with bacteria, in motility plates inoculated with the *ΔblsA* mutant and incubated at the indicated temperatures. Three independent experiments were performed in each case. The area of plates covered with bacteria was measured with ImageJ (NIH), and then the percentage of plate coverage was calculated. The mean ± SEM is informed. Asterisks indicate significant differences in light compared to dark conditions, as indicated by *t*-test (*p* < 0.01).

To determine the critical temperature of photoregulation of motility in *A. baumannii*, i.e., the temperature at which photoregulation is lost, we monitored motility at different temperatures from 18 to 35°C. Figures 1A,B show representative motility assays for ATCC 17978 at different temperatures from 18 to 35°C under both blue light illumination and dark conditions. It can be observed that bacterial motility was only inhibited between 18 and 24°C under blue light illumination, while photoregulation was lost above 24°C and the bacteria moved throughout the plates at this condition. In the dark, the bacteria spread throughout the plates at all temperatures. Hence, the above results indicate a dramatic change in *A. baumannii* photoregulation with temperature, with a sharp threshold between 24 and 25°C. It is noteworthy that below 18°C, we were not able to test motility at this condition, as bacterial growth was inhibited.

We also studied motility in different illumination and temperature conditions for the *ΔblsA* mutant. Contrary to the wild type, this strain moved covering the whole plates both under blue light and darkness at all temperatures assayed, with the exception of 21°C under blue light, a condition at which the cells showed a slight decrease in motility compared to darkness (Figure 1C).

### *blsA* Expression Levels Follow Photoregulation of Motility in ATCC 17978

**FIGURE 2:**
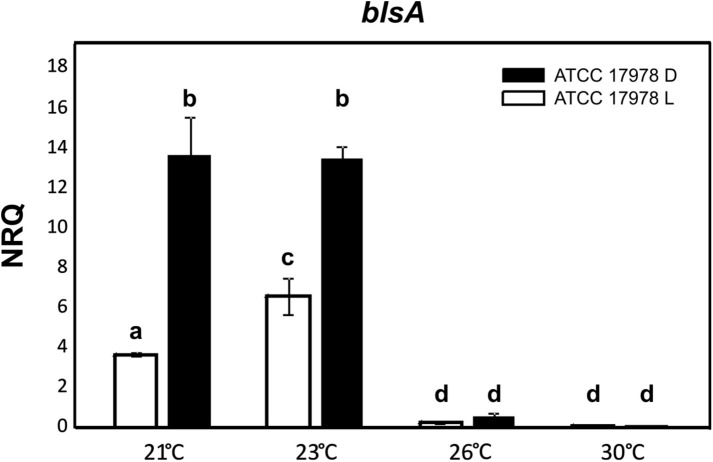
*blsA* expression levels follow photoregulation patterns. Estimation by RT-qPCR of the *blsA* expression levels in ATCC 17978 wild-type genetic background at different temperatures analyzed indicated in the *x*-axis, under blue light (L) or in the dark (D). The data shown are mean ± SD of normalized relative quantities (NRQ) obtained from transcript levels of *blsA* from cells recovered from motility plates incubated at the indicated temperatures under blue light or in the dark, measured in three biological replicates. Different letters indicate significant differences as determined by ANOVA followed by Tukey’s multiple comparison tests (*p* < 0.01).

*blsA* expression levels were analyzed in *A. baumannii* ATCC 17978 cells recovered from motility plates incubated at temperatures close to the critical values (24–25°C) under blue light or in the dark following procedures previously described (Müller et al., 2017). Figure 2 shows that *blsA* was expressed at 21 and 23°C, while its expression was almost negligible at 26 and 30°C. Moreover, at 21 and 23°C, *blsA* expression levels were higher in the dark than under blue light, in agreement with previous results (Mussi et al., 2010; Abatedaga et al., 2017). Globally, *blsA* expression levels followed the same photoregulation pattern observed for motility, with significant *blsA* levels at temperatures at which photoregulation occurred, while negligible levels were detected at non-photoregulatory temperatures.

### *blsA* Expression Levels Rapidly Respond to Illumination or Temperature Changes

**FIGURE 3:**
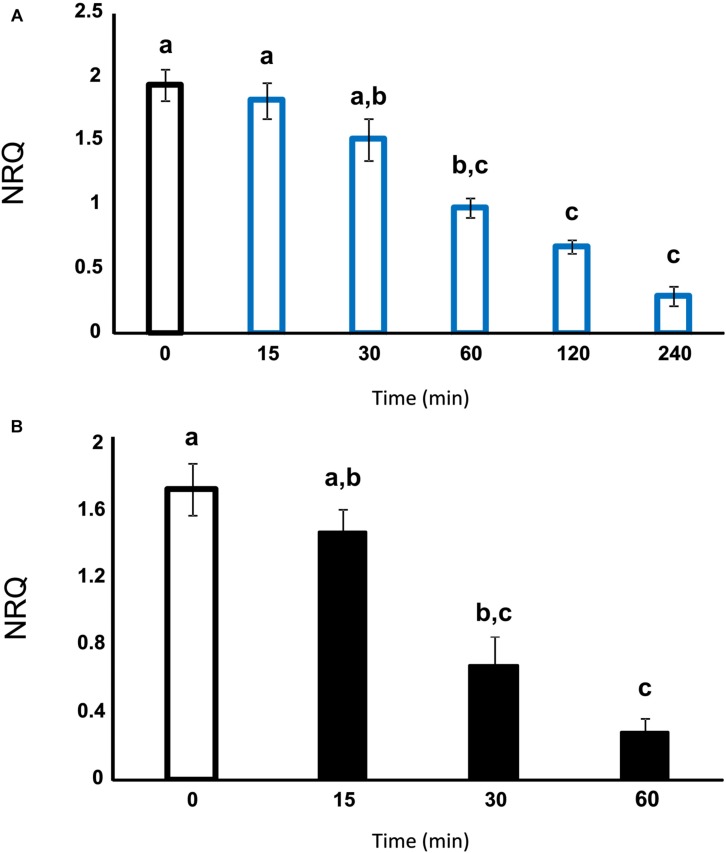
*blsA* expression levels rapidly respond to illumination or temperature changes. **(A)** Estimation by RT-qPCR of the *blsA* expression levels in ATCC 17978 cells grown until DO = 0.5 in LB at 24°C in the dark (D) (*t* = 0), and then switched to light conditions. **(B)** Estimation by RT-qPCR of the *blsA* expression levels in ATCC 17978 cells grown until DO = 0.5 in LB in the dark (D) at 24°C (*t* = 0), and then switched to 37°C. Samples were taken at the indicated times. The data shown are mean ± SD of normalized relative quantities (NRQ) obtained from transcript levels of *blsA* from cells incubated at the indicated temperatures under blue light or in the dark, measured in three biological replicates. Different letters indicate significant differences as determined by ANOVA followed by Tukey’s multiple comparison tests (*p* < 0.05).

Next, we studied the short-term response of the photoreceptor expression levels to light or temperature changes. As it can be observed in Figure 3, *blsA* expression levels significantly reduced when exponentially growing ATCC 17978 cultures adapted to dark conditions at 24°C were suddenly changed to illumination conditions (Figure 3A). In fact, after 60 min of illumination, *blsA* expression levels were half of those registered at zero time, consistent with the previous observations that *blsA* levels are higher in the dark than under blue light (Figure 2). Moreover, when cultures adapted to dark conditions at 24°C were shifted to 37°C, *blsA* expression levels were more efficiently reduced, since the half-time was reached after approximately 30 min (Figure 3B). Overall, these results show that the *blsA* photoreceptor expression system responds to illumination and temperature conditions rapidly at the physiological level.

### Photoreceptor Levels Are Negligible Above the Critical Temperature

**FIGURE 4:**
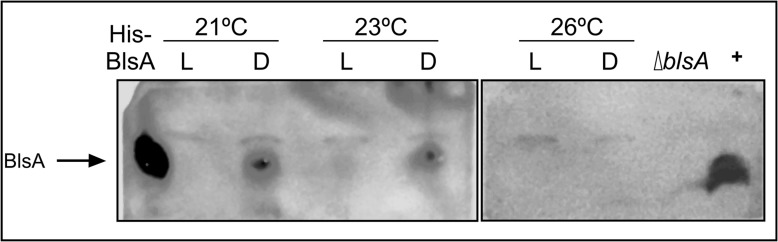
Effects of light and temperature on BlsA protein levels. Immunoblot analysis of protein samples from ATCC 17978 cells grown overnight in motility plates at 21, 23 or 26°C, in the presence of blue light (L) or in darkness (D), using purified antibodies directed against BlsA. Crude extracts corresponding to 350 μg of total proteins were loaded in each lane. His-BlsA refers to the purified recombinant BlsA protein, expressed as a His-tag fusion. (+) corresponds to the *ΔblsA* strain harboring plasmid pWHBlsA, which overexpresses a wild type copy of *blsA* directed by its own promoter.

Western blot analyses directed against BlsA (Figure 4) were in agreement with expression analyses at the RNA level. BlsA protein levels were detected at 21 and 23°C, being much higher in the dark than under blue light. Interestingly, BlsA levels were not detectable at 26°C both under blue light and in the dark. As controls, we have included the His-tagged recombinant BlsA (Mussi et al., 2010), as well as an extract of the *ΔblsA* mutant harboring plasmid pWHBlsA, which expresses a wild type copy of *blsA* directed by its own promoter.

### BlsA Photocycle Efficiency Is Compromised at 25°C and Practically Absent Above 28°C

**FIGURE 5:**
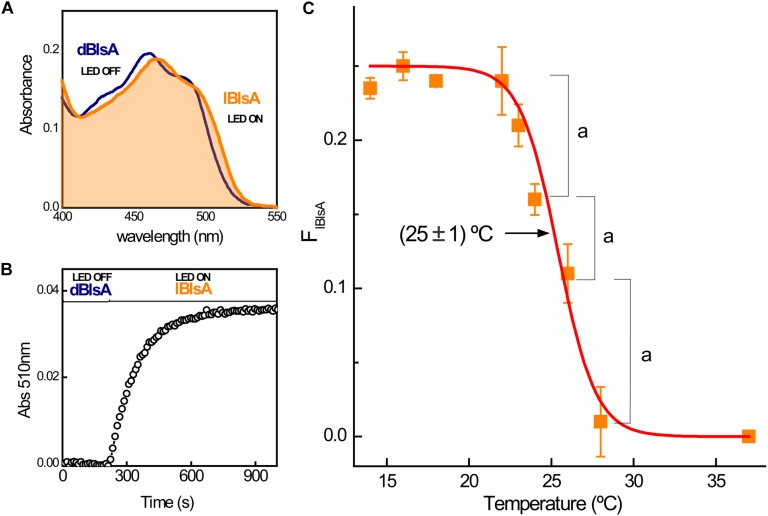
BlsA photoactivation efficiency (Φ_lBlsA_) versus temperature showing an inflection point at 25°C. **(A)** UV-vis absorbance changes produced for the dark-adapted BlsA, dBlsA turns into the light-adapted state, lBlsA by blue light illumination (Led Royal Blue, see Methods) at 14°C. **(B)** Kinetic profile for the lBlsA formation registered at 510 nm. **(C)** Variation of Φ_lBlsA_ with temperature, showing an inflection point at 25 ± 1°C. The data shown are mean ± SD of two independent measurements from two different protein productions. Letter (a) represents significant difference (*p* < 0.05) between the indicated temperature points.

To understand how temperature affects BlsA photoactivity, we analyzed the temperature-dependency of the BlsA photoactivation quantum yield (Φ_*lBlsA*_), since this value reflects the impact of temperature in the photoreceptor integrating all structural and local protein changes. Φ_*lBlsA*_, defined as the ratio of the light-adapted state of BlsA (lBlsA) concentration relative to the number of photons absorbed, was determined between 14 and 37°C by measuring the time-dependent UV-vis absorbance changes elicited on the dark-adapted BlsA (dBlsA) after blue light illumination to form the lBlsA state (Figures 5A,B; Abatedaga et al., 2017). Figure 5C shows the sigmoidal-like variation of Φ_*lBlsA*_ with temperature, with a maximum value of 0.24 ± 0.01 below 22°C. By increasing temperature above 22°C, Φ_*lBlsA*_ decreases significantly reaching non-detectable photoactivation at *T* > 28°C, suggesting that dBlsA is not active above this temperature. A sigmoidal fit of the data using a Boltzmann function indicates an inflection point at 25 ± 1°C. This value may represent the critical temperature for the dBlsA state, in which the sum of all conformational changes in the protein become important enough to alter its response to light (measured as photoactivation efficiency), leading to a complete loss of function above 28°C.

### Chromophore Binding Site Evidences Structural Instability With Temperature Increments

**FIGURE 6:**
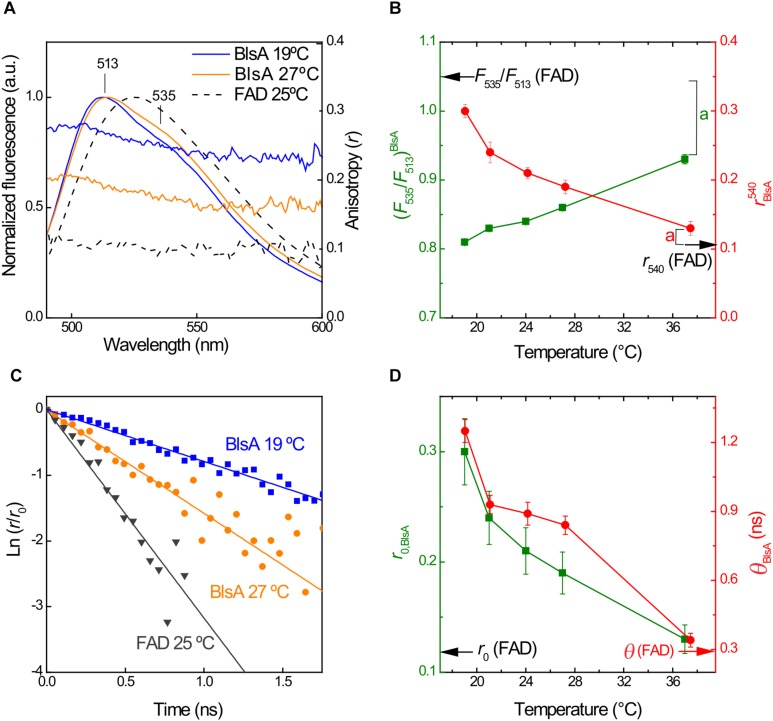
Temperature affects rigidity of the environment surrounding FAD in dBlsA. **(A)** Normalized fluorescence emission spectra of dBlsA at 19 and 27°C and FAD at 25°C in air-saturated buffer solution obtained by excitation at 460 nm. **(B)** Temperature dependence of the fluorescence intensity ratio between 535 nm and 513 nm (*F*_535_/*F*_513_) and steady-state anisotropy at 540 ± 20 nm (*r*_540_) for dBlsA. Arrows indicate the respective values for FAD and the respective steady state anisotropy. **(C)** Logarithmic first-order plots of the fluorescence anisotropy decays of dBlsA at 19 and 27°C monitored at 510 nm and FAD at 25°C monitored at 525 nm. **(D)** Temperature dependence of the initial anisotropy (*r*_0_) and the rotational correlation time (θ) for the cofactor in dBlsA. The data shown are mean ± SD of two independent measurements from two different protein productions. Letter (a) represents significant difference (*p* < 0.05) between dBlsA and free FAD at the indicated temperature points **(B,D)**.

Figure 6A compares the steady-state fluorescence emission and anisotropy spectra observed by excitation at 460 nm of dBlsA at 19 and 27°C with those of free FAD in the same buffer at 25°C. At 19°C, the vibrational structure of the emission fluorescence spectrum of dBlsA shows a maximum at 513 nm and a shoulder at 535 nm. This feature is gradually lost by a discrete red shift of the maximum when temperature increases, while shoulder disappearance mostly due to a decrease in its intensity without changes in its position. The variation of the ratio F_535_/F_513_ evidences this effect. Also, the steady-state anisotropy of the cofactor in dBlsA solutions is strongly decreased (Figure 6B). Moreover, the emission anisotropy was dependent on wavelength, being larger at the blue edge of the fluorescence spectrum. These results indicate an inhomogeneous population of emitters. However, for free FAD in the buffer solution, no dependence with temperature was observed, either in emission spectral shape or anisotropy, and the constant values are indicated with arrows at the respective *y*-axis of Figure 6B. The anisotropy decay monitored at 510 nm was also analyzed as a function of temperature, and in all cases they were fitted accordingly to the first-order Eq. (2) (Figure 6C), usually related to the depolarization of a spherical rotor (Lakowicz, 2006). It was observed that the increment of temperature decreases in the same proportion both for the initial anisotropy *r*_0_ and the rotational correlation time θ (Figure 6D). In fact, the trend is that at high temperatures, e.g., 37°C, both *r*_0__,BlsA_ and θ_*BlsA*_ are similar to the values for FAD.

### Intrinsic Fluorescence of BlsA Is Not Affected by Temperature

In order to further explore temperature-dependent conformational modifications in the secondary and tertiary structure of dBlsA, the intrinsic steady-state fluorescence of dBlsA was also studied by excitation at 295 nm, since dBlsA contains two Trp residues, i.e., Trp78 and Trp92 (Supplementary Figures S1A,B).

The intrinsic fluorescence spectrum of the protein was then practically constant in the temperature range studies (Supplementary Figure S1), with a blue-shifted emission maximum and narrower full width half-maximum (FWHM) than those for free Trp in buffer solution, respectively. This indicates that the nano-environment surrounding the Trp residues of BlsA did not change with the temperature. These results indicate that both Trp residues are partially buried in the protein regardless of the temperature, sensing at all times a less polar environment than in the aqueous buffer, as confirmed by the BlsA homology model (pdb template: 2HFN), where both Trp78 and Trp92 are partially protected from the solvent by nearby residues (Supplementary Figure S1B).

Hence, it can be concluded that the overall tertiary structure of the protein is conserved despite increments in temperature, in contrast to the spectroscopic changes observed with temperature for the cofactor due to discrete changes in the FAD-BlsA specific interaction in the active site.

### Size Particle Distributions Confirm the Formation of BlsA Aggregates With Temperature

**FIGURE 7:**
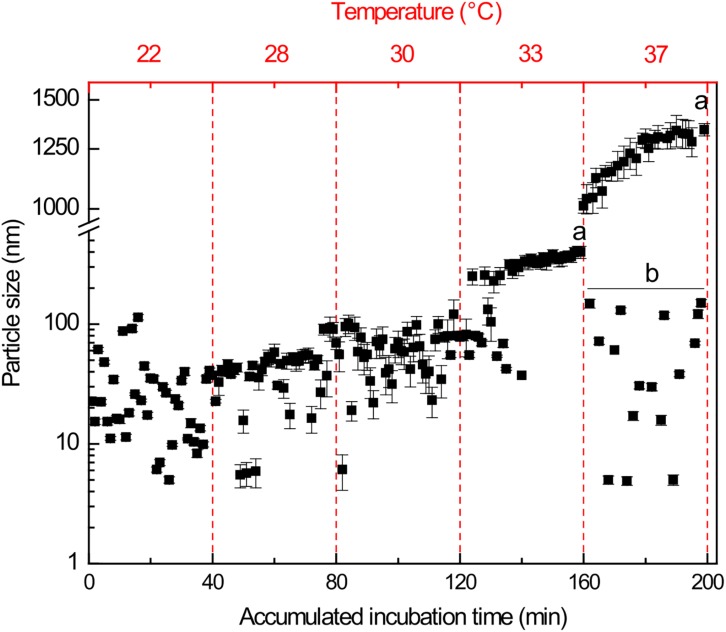
Particle size distribution of oligomeric states of dBlsA determined by DLS. The data shown are mean ± SD of two independent measurements from two different protein productions. Letter (a) represents significant difference (*p* < 0.05) between the first and last timepoint at any given temperature. Letter (b) represents significant difference (*p* < 0.05) between the mean of all values at the upper and the lower particle population (line).

Particle size distributions (PSD) determined by DLS measurements were followed during the incubation of dBlsA solutions during 40 min at each of the following temperatures: 22, 28, 30, 33, and 37°C (Figure 7). As temperature increases, there is a stepwise particle size increment, e.g., 20 ± 15 nm, 45 ± 10 nm; and 71 ± 24 nm at 22, 28, and 30°C, respectively. At 33 and 37°C the aggregation process is accelerated during the incubation period and much larger polydisperse aggregates are detected, e.g., 336 ± 65 nm and 1150 ± 150 nm, respectively. In fact, at 37°C a fraction of low size particles of 88 ± 40 nm was also detected. These results confirm the existence of protein aggregation effects concomitant with solution instability and the light inactivation of dBlsA.

## Discussion

Blue light exerts a global influence on *A. baumannii* through the BlsA photoreceptor. Disclosing the mechanism of BlsA functioning is therefore essential to understand an important aspect of this pathogen’s physiology. The evidence accumulated in this work, including physiological, gene expression and protein analysis, indicates that *blsA* expression and the corresponding photoreceptor levels fall above 24°C, reaching negligible levels. This is consistent with the lack of the photo-regulation observed for motility at these temperatures. It has been recently reported that motility depends on the pilus assembly system PrpABCD at 24°C, with differential expression of *prpA* in response to light in a BlsA-dependent manner in *A. baumannii* ATCC 17978 (Wood et al., 2018), confirming our previous results showing dependence on BlsA of this phenotype (Mussi et al., 2010). On the contrary, no photoregulation of motility was observed in the wild type cells at 37°C, while *prpA* expression showed no dependence on light at this temperature (Wood et al., 2018). Whether other yet unknown systems could also eventually contribute to motility at higher temperatures, the *prpABCD* system coding for type I pili governs motility and the response to light observed through BlsA in *A. baumannii* ATCC 17978 wild type cells (Wood et al., 2018). This notion is further supported by our data using the *ΔblsA* mutant, which show that this photoreceptor is the main responsible for the photoregulation of motility observed. Whether the reduction of motility perceived under blue light at 21°C corresponds to an uspecific effect of light on the motility machinery, or rather there exists another photosensory system operating, the data still show that BlsA is the major photoreceptor involved in the response. The overall data indicate that BlsA is a photoreceptor governing the response to blue light at low-moderate temperatures, i.e., *T* < 24°C, and the main line of control of its functioning is the regulation of its expression levels in the cell.

Previous work regarding BlsA functioning are in agreement with this notion. In this sense, BlsA has been shown to interact with transcriptional regulators such as Fur in a light dependent manner at 24°C but not at 30°C, and photoregulation of iron uptake itself occurs at 24°C but not at 30 or 37°C in *A. baumannii* ATCC 19606 (Tuttobene et al., 2018). Similar temperature-dependent modulation by light of acetoin catabolism has also been described for BlsA and the repressor AcoN in *A. baumannii* ATCC 17978 (Tuttobene et al., 2019b).

In our previous work, we demonstrated by monitoring the UV-vis absorbance changes at the red-edge of the flavin absorption band (see Figures 5A,B) that the efficacy of the photoconversion of the dark adapted dBlsA to form the lBlsA state was dependent on the displacement of FAD from its correct position inside the nanocavity active site (Abatedaga et al., 2017). In this condition, the conserved Tyr7 is very close to the isoalloxazine ring of the flavin to produce the ultrafast electron-transfer and subsequent proton-transfer processes between the cofactor to the residue generating the reduced semiquinone radical of FAD (FADH^⋅^). The efficiency of this process is measured by the quantum yield of photoconversion of the lBlsA state (Φ_*lBl*__*s*__*A*_), which falls from 0.22 to zero as the temperature increases above 22°C (Figure 5C). This result was accompanied with the progressive loss of anisotropy and red-shifting of the fluorescence of the cofactor in dBlsA with the temperature (Figure 6), linking the photo-regulation of BlsA with the environmental properties of the active site. In fact, the relatively low value of the rotational correlation time θ_*BlsA*_ = 1.25 ns at 19°C indicates that the light induced formation of FADH^⋅^ produces an efficient depolarization channel of the cofactor, since a 10-times larger value of θ can be expected for a globular protein of approximately 20 kDa as dBlsA (Lakowicz, 2006). It is interesting to note that as the nanoenvironment of FAD is changed with temperature, the intrinsic fluorescence of dBlsA reported by the Trp78 and Trp92 residues (Supplementary Figure S1A) indicated that no significant changes of the protein structure occurred. From the BlsA homology model both Trp residues are located in opposite sides of the molecule and outside the FAD binding site (Supplementary Figure S1B). This suggests that the BlsA side-containing FAD is more sensitive to increments in temperature than any other region of BlsA.

Moreover, besides the environmental rearrangement around the cofactor, temperatures above 30°C play a role on the protein aggregation process (Figure 7). Precipitation of BlsA above 30°C, which can be seen after centrifugation as a white pellet (Abatedaga et al., 2017) correlates with the presence of macroaggregates (HD > 80 nm) as determined with DLS. This aggregation process appears to be dependent on temperature. Interestingly, from DLS data at 37°C, soluble microaggregates corresponding to non-functional low oligomeric forms (HD ∼80 nm) are present and may well confirm that the protein remaining in solution regenerates the functional oligomeric forms, once the temperature is restored to 24°C or lower temperatures, recovering the photocycling activity, as previously shown (Abatedaga et al., 2017). All these data suggest that the conformational changes are somewhat reversible but limited by the temperature exposure time, the longer the exposure time at temperatures > 30°C, the higher amounts of aggregates are formed and the larger the particle sizes. This behavior has been described for other proteins such as bovine serum albumin (BSA) (Su et al., 2008).

It should be noted that all the evidence gathered indicates that BlsA is a photoreceptor that functions both in the dark and under blue light, and therefore “the signaling state” frequently referred to as the state acquired by photoreceptors upon illumination does not apply here. Indeed, it has been recently shown that BlsA binds to and likely captures transcriptional repressors such as Fur in the dark at 23°C (Tuttobene et al., 2018), but also other set of transcriptional repressors, such as AcoN, under blue light (Tuttobene et al., 2019a). This differential ability to bind several transcriptional regulators, both under blue light or in the dark in a temperature dependent manner, is probably related to the differential oligomerization states acquired by BlsA under blue light/dark or induced by different temperatures. We are currently working on this matter to provide more insights in the future.

In summary, BlsA modulation by temperature occurs on two levels: one at cellular level, controlling the photoreceptor expression and production in the cells, which is drastically reduced above the critical temperature. The second modulation point is at the protein level, involving a deleterious effect of temperature above the critical point in the interaction of FAD and the residues surrounding it in its nanocavity with a concomitant loss of photoactivity, and general conformational changes that lead to protein aggregation.

## Data Availability

All datasets generated for this study are included in the manuscript and/or the Supplementary Files.

## Author Contributions

AG, LV, IA, CÁ, PJ, and CP performed the experiments. LV collaborated in writing the manuscript. CÁ analyzed the experiments. MM, IA, LV, and CB designed the experiments and wrote the manuscript. MM and CB provided funding.

## Conflict of Interest Statement

The authors declare that the research was conducted in the absence of any commercial or financial relationships that could be construed as a potential conflict of interest.

## Supplementary Material

FIGURE S1 Intrinsic fluorescence emission is not affected by temperature. **(A)** Normalized emission spectra of dBlsA, from 19 and 37°C by excitation at 295 nm. Inset: dBlsA Full Width at Half Maximum (FWHM) and maximum emission wavelength (λ_*max*_) versus temperature. **(B)** Detail of BlsA homology model (template:2HFN) showing relative positions of Tyr7, Gln51, Trp78 and Trp92 to the FAD molecule.
