# Supplementary figures and images for: BlsA Is a Low to Moderate Temperature Blue Light Photoreceptor in the Human Pathogen Acinetobacter baumannii
Source: Front Microbiol. 2019 Aug 21;10:1925. doi: 10.3389/fmicb.2019.01925 (PMC6712483; doi:10.3389/fmicb.2019.01925)

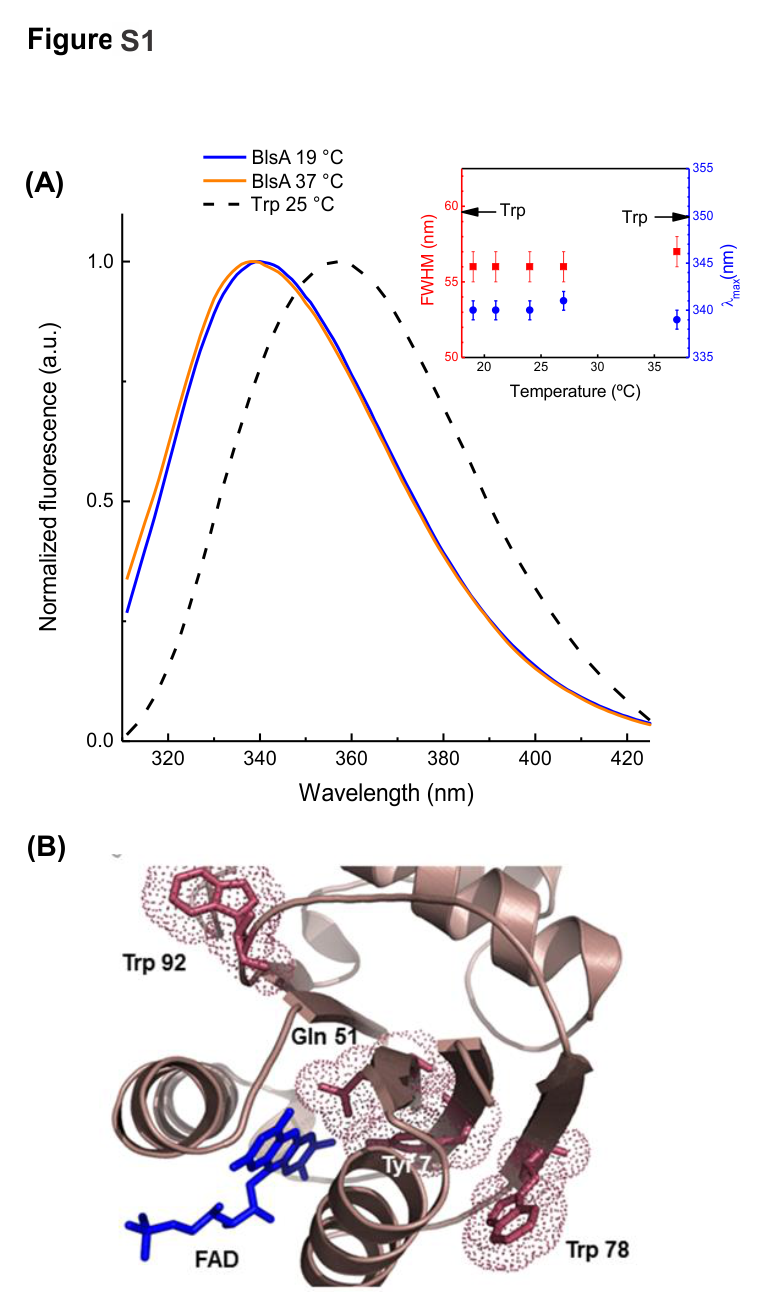

Supplement: FIGURE S1 — Intrinsic fluorescence emission is not affected by temperature. (A) Normalized emission spectra of dBlsA, from 19 and 37°C by excitation at 295 nm. Inset: dBlsA Full Width at Half Maximum (FWHM) and maximum emission wavelength (λmax) versus temperature. (B) Detail of BlsA homology model (template:2HFN) showing relative positions of Tyr7, Gln51, Trp78 and Trp92 to the FAD molecule. [file Image_1.TIF]
